# Extremely Cost‐Effective and Efficient Solar Vapor Generation under Nonconcentrated Illumination Using Thermally Isolated Black Paper

**DOI:** 10.1002/gch2.201600003

**Published:** 2017-01-30

**Authors:** Zhejun Liu, Haomin Song, Dengxin Ji, Chenyu Li, Alec Cheney, Youhai Liu, Nan Zhang, Xie Zeng, Borui Chen, Jun Gao, Yuesheng Li, Xiang Liu, Diana Aga, Suhua Jiang, Zongfu Yu, Qiaoqiang Gan

**Affiliations:** ^1^ Department of Electrical Engineering The State University of New York at Buffalo Buffalo NY 14260 USA; ^2^ Department of Materials Science Fudan University Shanghai 200433 China; ^3^ Department of Environmental Science and Engineering Fudan University Shanghai 200433 China; ^4^ Department of Chemistry The State University of New York at Buffalo Buffalo NY 14260 USA; ^5^ Department of Electrical and Computer Engineering University of Wisconsin Madison WI 53705 USA

**Keywords:** carbon materials, solar‐to‐heat conversion, solar vapor generation, thermal isolation

## Abstract

Passive solar vapor generation represents a promising and environmentally benign method of water purification/desalination. However, conventional solar steam generation techniques usually rely on costly and cumbersome optical concentration systems and have relatively low efficiency due to bulk heating of the entire liquid volume. Here, an efficient strategy using extremely low‐cost materials, i.e., carbon black (powder), hydrophilic porous paper, and expanded polystyrene foam is reported. Due to the excellent thermal insulation between the surface liquid and the bulk volume of the water and the suppressed radiative and convective losses from the absorber surface to the adjacent heated vapor, a record thermal efficiency of ≈88% is obtained under 1 sun without concentration, corresponding to the evaporation rate of 1.28 kg (m^2^ h)^−1^. When scaled up to a 100 cm^2^ array in a portable solar water still system and placed in an outdoor environment, the freshwater generation rate is 2.4 times of that of a leading commercial product. By simultaneously addressing both the need for high‐efficiency operation as well as production cost limitations, this system can provide an approach for individuals to purify water for personal needs, which is particularly suitable for undeveloped regions with limited/no access to electricity.

## 1 Introduction

Efficient solar energy‐to‐heat conversion for vapor/steam generation is essential for various applications ranging from large scale absorption chillers, desalination systems to compact and portable applications including drinking water purification and sterilization systems.^1^, ^2^, ^3^, ^4^, ^5^ Conventional solar steam generation techniques usually rely on costly and cumbersome optical concentration systems to heat a bulk liquid.^6^ Even though some highly absorbing materials are utilized to enhance solar absorption, such as charcoal,^7^ sponge,^8^ or cotton cloth,^9^ the energy conversion efficiency is still relatively low (e.g., 30–40%^10^) due to the heat dissipation in the entire liquid volume. Therefore, there is a significant need to develop more efficient, self‐powered, and highly portable solar energy harvesting systems for vapor/steam generation. Low‐cost and broadband light absorbing micro/nanomaterials show promise in this regard.

In recent years, plasmonic nanoparticles (NPs) and their assemblies have been widely studied because of their unique light and heat localization properties. In particular, it was revealed that the localized heat effect can be used for new solar vapor/steam generation that cannot be addressed using conventional technologies that heat the entire fluid volume. For instance, plasmonic metallic NPs^11^, ^12^, ^13^ and nanorods^14^, ^15^ dispersed in aqueous solutions can generate vapor bubbles. However, due to limited solar absorption bands, the resulted solar thermal conversion efficiencies of these earlier works are relatively low. For example, it was reported that Au NPs dispersed in water obtained a solar thermal conversion efficiency of 24% (i.e., only 24% of the solar energy was transferred to generate vapor).^16^ To overcome this bandwidth limitation, broadband dark metallic nanostructures (e.g., Au and Al‐based NPs^17^, ^18^, ^19^, ^20^) were developed to enhance the overall solar‐to‐heat conversion efficiency (e.g., 57.3% under illumination of 20 kW m^−2^ (i.e., 20 sun concentration)^17^ and 92.6% under illumination of 6 kW m^−2^ (i.e., 6 sun concentration) using ultrabroadband black gold membrane structures,^18^ 77.8% under illumination of 4.5 kW m^−2^ (i.e., 4.5 sun concentration) using airlaid‐paper‐based Au NP structure^19^). However, the intrinsically high cost of Au‐based nanomaterial (e.g., retail price of $395 mg^−1^ for Au nanoshells^21^) is a significant bottleneck for practical applications using these systems. This is especially true when absorbing NPs are dispersed throughout the bulk of a liquid (e.g., ref. 16) and a significant number is effectively wasted due to absorption and scattering of the incident light by the NPs above.

To overcome this issue, floating substrates such as carbon foam,^22^ paper,^19^ and nanoporous anodic alumina^17^, ^18^, ^20^ have been employed to localize the absorbing material at the surface of water for more efficient and cost‐effective solar steam generation. In these platforms, the substrates functioned as thermally insulating layers that reduce the heat transfer between the vaporization region (i.e., the water surface) and the bulk liquid. Due to the capillary action of these porous supports, localized evaporation was realized with improved thermal efficiencies (e.g., 64% under 1 kW m^−2^ illumination using exfoliated graphite on carbon foam^22^). Additionally, it was reported that the use of solar concentrators further improved the thermal efficiencies of these systems, up to 85–90% (e.g., ref. 17, 18, 19, 20, 22). However, in order to achieve these high efficiencies, these platforms still require specialized fabrication of highly absorbing, structured nanomaterials (e.g., black gold or aluminum NPs on nanoporous anodic alumina^17^, ^18^, ^20^) and/or porous hydrophilic supports (e.g., porous carbon foams at the retail price of ≈$1.5 in.^−3^ 23), as well as costly solar concentrating systems. These requirements impose prohibitively high costs for practical applications over large areas.

In this work, we report an efficient carbon‐based solar vapor generation system based on carbon‐coated paper (CP) affixed to expanded polystyrene (EPS) foam. Due to the superior absorption, heat conversion, and insulating properties of our CP‐foam structure, most of the absorbed energy can be used to evaporate surface water with significantly reduced thermal dissipation compared with previously reported architectures.^24^, ^25^, ^26^ Remarkably, we realized a record solar thermal conversion efficiency of >88% under illumination of 1 kW m^−2^ with no solar concentration. Furthermore, seawater desalination was also demonstrated with reusable stable performance. By utilizing extremely low‐cost materials, and circumventing the need for solar concentrators, economically viable large area systems will be possible with no energy input required for operation. This prospect is particularly attractive for addressing global freshwater shortages, especially for individuals to purify water for personal needs (i.e., ≈2 L d^−1^) in developing regions.

## 2 Results

### 2.1 CP for Solar Vapor Generation

**Figure 1:**
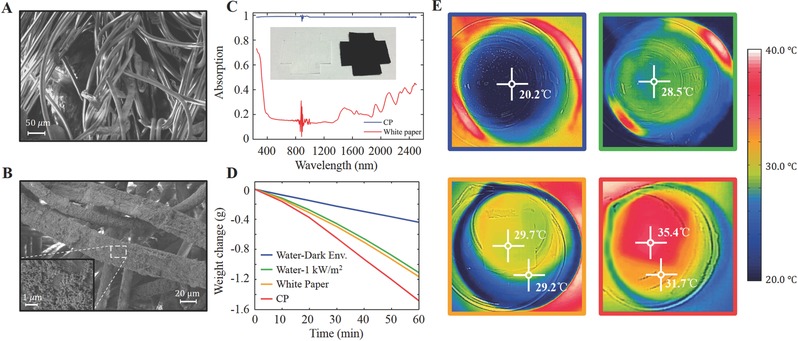
A) Scanning electron microscope (SEM) image of uncoated fiber‐rich paper. B) SEM image of CP under low and high magnifications (inset). C) The absorption spectra of uncoated white paper and CP measured by an integration sphere. Inset: Photograph of these two pieces of paper. D) Comparison of water weight change versus time under four different conditions: water in dark environment (blue line), water under 1 kW m^−2^ illumination (green line), floating white paper under 1 kW m^−2^ illumination (orange line), and floating CP under 1 kW m^−2^ illumination (red line). E) The surface temperature distribution of the four samples measured in (D), measured using a thermal imager: the upper left panel corresponds to the blue line; the upper right panel corresponds to the green line; the lower left panel corresponds to the orange line; and the lower right panel corresponds to the red line in (D).

In previously reported pioneering works based on porous materials (e.g., ref. 17, 18, 19, 20, 22), capillary force is essential to assist the enhanced vapor generation process since it is much easier to vaporize small droplet diffused into the pores than to heat and vaporize the bulk volume. In principle, hydrophilic porous materials are generally suitable for this purpose.^27^ In this work, we selected a fiber‐rich nonwoven paper (Texwipe TX609^28^) as our support since it is extremely low‐cost (i.e., retail price of ≈$1.05 m^−2^), chemical‐binder‐free, and has excellent water transport properties. Its microstructure is shown in **Figure** **1**A, consisting of 10–20 μm wide paper‐fiber bundles. We then dye it using low‐cost carbon black powders (e.g., Sid Richardson Carbon & Energy Co., retail price of $2.26 lb^−1^; see Section S1 in the Supporting Information for fabrication details and stability/durability test results). As a result, the paper fibers were coated with carbon nanoparticles, as shown in Figure 1B. The direct comparison between the white paper and the carbon‐coated paper is shown in the inset of Figure 1C. The optical absorption of the CP is very strong with the average absorption of ≈98% throughout the visible to near IR domain (from 250 nm to 2.5 μm, measured by a spectrophotometer equipped with an integration sphere, Shimadzu UV‐3150). This strong broadband optical absorption is particularly promising for low‐cost solar‐to‐heat conversion. It should be noted that although the latest reported Al‐nanoparticle structure is also inexpensive if implemented in yield productions,^20^ the inflammability of 5–30 nm sized Al‐NPs imposes a potential safety issue (see the Safety Data Sheet of Al NPs,^29^ code H261^30^). Therefore, the proposed CP structures are also superior since they are environmentally benign and safe to handle during production.

To demonstrate the baseline for solar vapor generation performance, we first performed a direct comparison under several different conditions as shown in Figure 1D (see Section S2 in the Supporting Information for experiment details). In this experiment, the open area of the beaker is 35.3 cm^2^, containing ≈165 g water. In the dark environment (i.e., at room temperature of 21 °C and humidity of 10%), the water weight loss is 0.44 g h^−1^. Therefore, the average evaporation rate in the dark environment is 0.125 kg (m^2^ h)^−1^, which will be subtracted from all subsequent measured evaporation rates to eliminate the effect of natural water evaporation. Under the solar illumination using a solar simulator (Newport 69920 with the solar intensity of 1 kW m^−2^, i.e., AM1.5), the weight loss increased to 1.11 g h^−1^. After that, we put a 4 × 4 cm^2^ white paper and a 4 × 4 cm^2^ CP on top of the water surface, the weight change increased to 1.16 and 1.48 g h^−1^, respectively. To interpret the weight change difference, we employed a portable thermal imager (FLIR ONE) to characterize the temperature of these samples. The thermal imaging characterization was confirmed by a direct measurement using a thermocouple sensor probe (see Section S3 in the Supporting Information), indicating a reasonable accuracy (i.e., ≤0.4 °C in the 33–35 °C range). As shown in Figure 1E, the CP surface temperature increased to the highest number of 35.4 °C due to the enhanced solar‐to‐heat conversion. However, this heating effect is not well isolated from the bulk water (i.e., the bulk water was heated to 31.7 °C), resulting in the inefficient vapor generation effect. One can see that the water evaporation speed with the CP is 1.33 times higher than that of pure water under the 1 kW m^−2^ solar illumination, which is only an incremental improvement. Next, we will discuss the thermal‐isolating strategy to confine the heating effect at the top surface for more efficient vapor generation.

### 2.2 Efficient Vapor Generation Using Thermally Isolated CP

**Figure 2:**
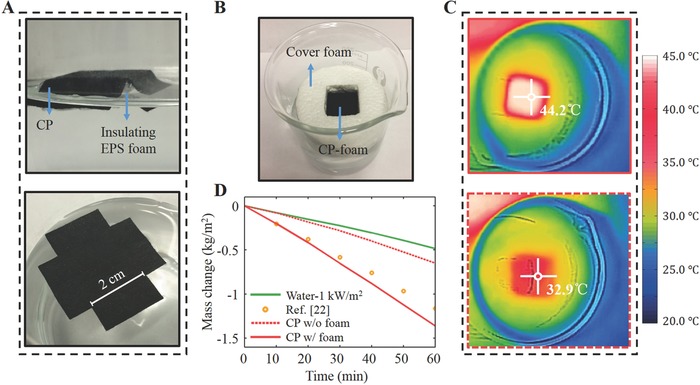
A) Photographs of a CP with (upper panel) and without the insulating EPS foam (lower panel) floating on top of water. B) Photograph of the CP‐foam structure with cover foam to eliminate evaporation from the water surface surrounding the CP‐foam structure. C) Surface temperature distribution of a CP with (upper panel) and without the insulating EPS foam (lower panel) floating on the water. D) Comparison of water mass change due to evaporation versus time under four different conditions: water under 1 kW m^−2^ (green line), exfoliated graphite on carbon foam from ref. 22 (orange circles), CP without insulating foam (red dashed line), and CP with insulating foam (red solid line).

One of the most attractive features claimed by previously reported nanomaterials for solar‐vapor generation is the surface heating effect with no need to heat the bulk volume of the water (e.g., ref. 17, 18, 19, 20, 22). According to pioneering studies employing carbon foams,^22^ nanoporous alumina,^17^, ^18^, ^20^ and floating paper,^19^ porous supports transport small water droplets to the upper surface directly through the structure of the support. Although they are also designed to serve as thermal insulators, the finite thickness, large contact area, and fluid transport of the porous substrates lead to relatively poor thermal insulation performance (e.g., the thermal conductivities are 0.49 W (m K)^−1^ in ref. 19 and 0.426 W (m K)^−1^ in ref. 22], respectively). Therefore, a better thermal isolation will improve the solar vapor generation performance. In this work, we propose a better strategy to make full use of the capillary force of the porous paper to draw fluid up around the support rather than through it, and thus minimize the thermal loss to the bulk fluid below. As shown by the upper panel in **Figure** **2**A, we inserted a 6 mm thick EPS foam slab under the CP to thermally isolate the porous paper from the bulk water. The thermal conductivity of this EPS foam is 0.034–0.04 W (m K)^−1^,^31^ one of the lowest thermal conductivities available among extremely low‐cost materials. In this configuration, the only contact area between the water and CP is at the edges of the porous paper (i.e., a line contact). This significantly reduces the region of fluid transport compared to placing the paper^19^ or carbon foam^22^ directly on the water surface (see the lower panel in Figure 2A). In this case, the paper contacting the water along the sides of the EPS foam transports the water droplets to the upper surface to facilitate evaporation. It should be noted that during testing, the upper surface of the CP was always wet, indicating that this reduction in transport area does not limit the evaporation rate of the system. A more detailed characterization of the liquid transportation capability of the CP is shown in Section S4 (Supporting Information).

To eliminate the water evaporation from other open areas, the surrounding exposed water surface was covered with EPS foam with a square hole for the CP (Figure 2B). To demonstrate the thermal isolation effect, we then characterized the surface temperature with and without the EPS foam under the CP, as shown in Figure 2C. Under the solar light illumination with the intensity of 1 kW m^−2^, the upper surface temperature of the CP increased from 32.9 °C (lower panel) to 44.2 °C with the EPS foam insulation (upper panel). The vapor generation performance is shown in Figure 2D. One can see that the water mass change is improved to 1.28 kg (m^2^ h)^−1^, which is 3.0 times greater than that of the pure water case and 2.0 times greater than that of CP without EPS foam isolation. This evaporation rate is better than the best reported data under 1 sun illumination with no solar concentration using exfoliated graphite (i.e., circles taken from Figure 2D of ref. 22). In principle, one would only need a ≈0.2 m^2^ structure to produce 2 L of freshwater to meet an individual's daily needs assuming 8 h of nonconcentrated solar illumination. Solar concentration will enhance this generation rate further, as will be discussed next.

### 2.3 High Solar Thermal Conversion Efficiency

**Figure 3:**
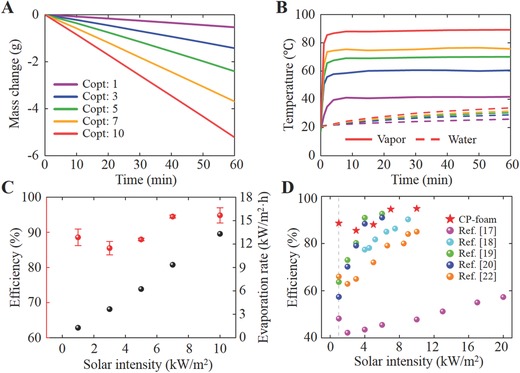
A) The water mass change as a function of time under 1, 3, 5, 7, and 10 times concentrated solar illumination, respectively. B) The temperature change as a function of time under 1, 3, 5, 7, and 10 times concentrated solar illumination, respectively. The solid lines represent vapor temperatures measured by a thermometer installed above the CP‐foam. The dashed lines represent bulk water temperatures measured under the foam, while line colors are as for the legend of (A). C) The solar thermal conversion efficiency (red dots) and corresponding evaporation rate (black dots) as a function of solar intensity. D) Direct comparison of solar thermal conversion efficiencies obtained by previously reported structures (data from refs. 17, 18, 19, 20, 22) and the CP‐foam.

In most previously reported works,^17^, ^18^, ^19^, ^20^, ^22^ the sample surface is always wet, indicating that the performance is limited by surface temperature only. Therefore, the ultimate performance can be improved by introducing concentrated solar illumination. Next, we will analyze the vapor generation performance under moderate solar concentration conditions to better compare with previously reported nanostructures. In this experiment, an inexpensive planar PVC Fresnel lens (e.g., OpticLens, $2.39 per piece with the area of 26 cm × 17.8 cm) was employed to focus the incident light from the solar simulator. As shown in **Figure** **3**A, when the solar light was concentrated by 3, 5, 7, and 10 times, the water mass change was increased to 3.66, 6.24, 9.34, and 13.30 kg (m^2^ h)^−1^, respectively. To characterize the enhanced surface heating effect more accurately, we then employed two thermocouple sensor probes to measure the temperature of vapor and bulk water (see Figure S3 in the Supporting Information). As shown by solid curves in Figure 3B, the vapor temperature increased sharply within the first 3 min and reached a steady state after 10 min. In contrast, the temperature of bulk water increases slowly and continuously as shown by dashed lines in Figure 3B. To evaluate the solar‐vapor generation performance quantitatively, we then calculate the solar conversion thermal efficiency, η_th_, which is described by Equation (1), 22

(1) $$\eta_{\mathrm{th}}=\frac{\dot{m}h_{\mathrm{LV}}}{C_{\mathrm{opt}}q_{i}}$$

where $\dot{m}$ is the mass flux, *h*_LV_ the total enthalpy of liquid‐vapor phase change, *C*_opt_ the optical concentration, and *q*_i_ the normal direct solar irradiation (i.e., 1 kW m^−2^). Particularly, the calculation of the total enthalpy of liquid‐vapor phase change, *h*_LV_, should consider both the sensible heat and the temperature‐dependent enthalpy of vaporization (see Section S4 for details of this equation and calculation in the Supporting Information). Using Equation (1), we obtained the solar conversion thermal efficiency, η_th_, of 88.6% under 1 sun illumination, and 94.8% under 10 times solar concentration, as shown in Figure 3C. Compared with previously reported exfoliated graphite,^22^ Au NPs,^18^ and black gold membranes,^17^ this CP‐foam structure realized a very high solar thermal conversion efficiency especially under low optical concentration condition (see direct comparison in Figure 3D calculated by similar data processing procedures; see Section S5 in the Supporting Information for more details). It should be noted that since the reported CP structure does not require any special micro/nanofabrication process, the system is extremely low‐cost (cheaper than that of the concentrator) and amenable to scaling up over large or huge areas for real applications. Therefore, there is no need to employ large area solar concentrating systems for real applications.

In addition, this η_th_ actually describes the energy consumption in the vapor, and has two major components: the energy used for water‐to‐vapor phase change, and the energy used to heat the water/vapor. A larger η_th_ does not necessarily correspond to a higher vapor generation rate. For a given value of η_th_, a higher temperature of the generated vapor will actually result in a lower generation rate since more energy is used to heat the water. Therefore, in terms of solar vapor generation rate, it is necessary to analyze the theoretical upper limit and thermal loss channels in order to estimate the opportunity available for improvement.

### 2.4 Theoretical Upper Limit

The ideal condition for solar vapor generation is to convert liquid water to vapor at ambient temperature with no energy used to heat either the bulk or evaporated water. Radiative loss and convective loss are both assumed to be zero. In this case, based on our experimental conditions (i.e., at ambient temperature of 21 °C), the ideal vapor generation rate is 1.466 kg (m^2^ h)^−1^ assuming η_th_ = 1 and *h*_LV_ in Equation (1) is 2455.6 kJ kg^−1^ at ambient temperature.^32^ Based on this ideal vapor generation rate, we can straightforwardly estimate η_th_ obtained in our experiment, i.e., 1.28/1.466 = 87.3%, which only considers the energy used to produce vapor at room temperature. Detailed thermal loss mechanisms are automatically excluded in this simple estimation. However, this theoretical upper limit is unlikely realized since thermal losses are inevitable in these systems. Additionally, under 10× solar concentration, this theoretical maximum is 14.66 kg (m^2^ h)^−1^. Therefore, even if these theoretical upper limits can somehow be further approached using advanced (and likely expensive) nanomaterials in the near future, the opportunity for improvement is relatively limited. As a result, the more pressing issue in developing technologies for high performance solar vapor generation is cost, which is the primary advantage of our proposed structure and system.

### 2.5 Loss Channels

Recently, a new strategy was reported to demonstrate the close to 100 °C steam generation under 1 sun enabled by a floating structure with “thermal concentration.”^33^ A detailed thermal loss analysis was performed, revealing that radiative loss and convective loss are two major thermal loss channels in the solar vapor generation systems. The radiative and the convective losses per area are expressed by Equations (2) and (3), respectively

(2) $$P_{\mathrm{rad}}=\varepsilon \sigma \left(T_{2}^{4}\;-\;T_{1}^{4}\right)$$

(3) $$P_{\mathrm{con}}=h\left(T_{2}\;-\;T_{1}\right)$$

where ε is the emissivity of the CP (i.e., 0.98), σ the Stefan–Boltzmann constant (i.e., 5.67 × 10^−8^ W (m^2^ K^4^)^−1^), *T*_2_ the temperature at the surface of the CP, *T*_1_ the temperature of the adjacent environment, and *h* the convection heat transfer coefficient (assumed to be 10 W (m^2^ K)^−1^ 33). Using these two equations, it was estimated that the radiative loss from the 100 °C blackbody absorber surface to the ambient environment (20 °C) is ≈680 W m^−2^ and the convective loss is ≈800 W m^−2^. Following this theoretical estimation, when the absorber surface is 44.2 °C (our experimental observation), the radiative loss to ambient is ≈147 W m^−2^ and the convective loss is ≈232 W m^−2^, corresponding to a total of 37.9% energy loss (i.e., 14.7 + 23.2%). In this case, it seems that an efficiency ≈90% is impossible. But why can we observe a record high vapor generation rate under 1 sun?

**Figure 4:**
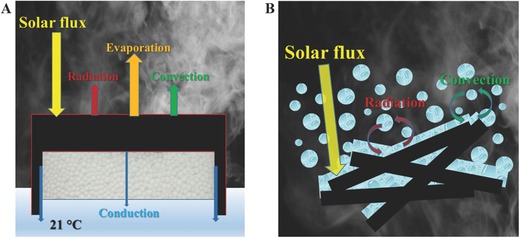
A) Energy balance and heat transfer diagram in the CP‐foam architecture during the vapor generation process. B) Zoom‐in diagram near the surface of the CP structure during the vapor generation process.

To interpret the unique features and physics of the proposed CP‐foam architecture, the thermal environment and heat transfer diagram is analyzed in **Figure** **4**A. First, the downward thermal radiation is suppressed. According to the previously reported experimental characterization, the reflection of a 3 mm thick EPS foam slice is in the range of 40–60% over the spectral region of thermal emission with ≈10% thermal radiation absorption.^34^ Therefore, under thermal equilibrium condition, the temperature of the EPS‐foam surface is very close to the bottom surface of the CP layer so that the downward radiative loss from the CP layer is significantly suppressed. In this case, the EPS foam employed in our system actually serves as a thermal radiation shield (in addition to its excellent thermal insulation characteristics), which is superior over previously reported double‐sided black systems (e.g., ref. 22, 35).

If we further analyze the microscopic thermal environment (Figure 4B), one can recognize that the CP surface is covered by a sheet of water and surrounded by heated vapor. The absorbed solar energy of the CP layer will first exchange thermal energy with water sheet and vapor in this small region rather than directly emit thermal radiation and exchange heat with the surroundings through the convection. In particular, in many reported experiments to identify the vapor temperature, a thermocouple was usually placed on top of the absorber surface (e.g., ref. 18, 20, 22, 33), further demonstrating that the top surface of the absorber is surrounded by heated vapor. Since the temperature of the adjacent environment on top of CP absorber is very close to the temperature of CP surface, the radiative and convective loss should be very small. For instance, according to Equations (2) and (3), the radiative loss from the 44.2 °C surface under 1 sun to the ≈41.6 °C vapor environment is ≈1.8% and the convective loss is only ≈2.6%. Most absorbed solar energy is still used to evaporate the water sheet on top of the absorber surface rather than being lost through these two channels. This is the major physical mechanism for the observed high vapor generation rate. This is also applicable to other reported solar vapor generation systems (e.g., ref. 17, 19, 22, 27, 36, 37, 38) since they are also covered by a film of water and/or surrounded by heated vapor. However, this physical mechanism was not detailed in previous reports.

More importantly, in a real enclosed solar steam system, the vapor cannot be released immediately and the environment inside the system is thermally isolated from the cooler surrounding environment. Furthermore, typical acrylic or glass slabs are opaque to mid‐infrared radiation. Consequently, thermal radiation cannot be emitted to the environment. Additionally, convective energy transfers are also largely suppressed when the internal environment is heated under near‐thermal equilibrium conditions. In this case, the radiative and convective losses in a real system should be even more negligible, demonstrating the potential to develop practical solar steam systems using extremely low‐cost materials. Intriguingly, in the latest report,^33^ the highest temperature of the generated steam was observed in a vapor chamber, demonstrating the accuracy of our proposed physical picture. In the next section, we will continue to demonstrate its application for seawater desalination, a process to remove salts and minerals to generate freshwater, representing a key solution to address the emerging water scarcity faced by this world.^3^, ^4^, ^5^

### 2.6 Performance for Solar Desalination and the Effect of the Bulk Water Temperature

**Figure 5:**
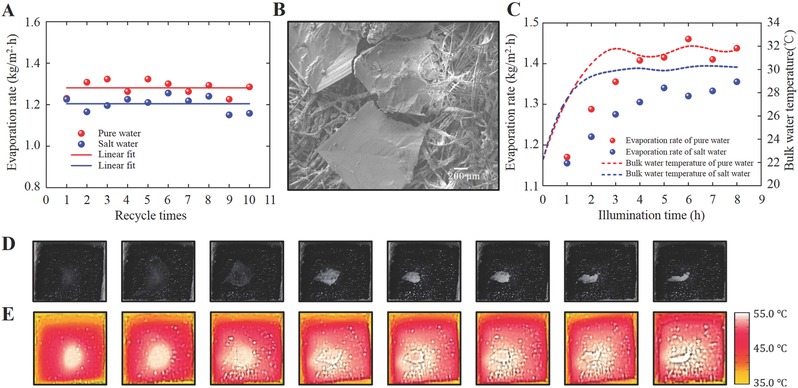
A) The evaporation rate of CP‐foam samples on salt water (blue spheres) and pure water (red spheres) as the function of cycle number. The two solid lines are guide for the eye to show the stable performance. B) The SEM image of a CP sample after 1 h evaporation in salt water. C) The evaporation rate of CP sample in salt water over an 8 h evaporation period as a function of illumination time. D) Photographs and E) thermal images of a CP‐foam on salt water at times corresponding to the blue spheres in Figure 5C.

Conventional desalination technologies are usually energy demanding (e.g., reverse osmosis membrane technology consumes ≈2 kW h m^−3^ 5) with serious environment costs. It was estimated that a minimum energy consumption for active seawater desalination is ≈1 kW h m^−3^,^3^ excluding prefiltering and intake/outfall pumping. Passive solar desalination technology is particularly attractive due to the electricity‐free operation with minimum negative impacts on the environment. To characterize the evaporation performance and reusability of our CP‐foam for desalination, here we prepared salt water with 3.5 wt% NaCl and performed the solar water evaporation experiment repeatedly. For each cycle, two CP‐foam samples were put on the surfaces of salt water and pure water, respectively, and illuminated under 1 kW m^−2^ for 1 h. After that, the CP samples were dried completely and reused for the next cycle. As shown in **Figure** **5**A, the evaporation rates of ten cycles in pure water and salt water are both stable (i.e., 1.2–1.3 kg (m^2^ h)^−1^), demonstrating the reliability of the proposed CP‐foam. Considering the excellent wet and dry strength and autoclavable features of the fiber‐rich nonwoven paper (Texwipe TX609^28^), it is particularly attractive for long term solar desalination application, which is still under test.

Noticeably, after the 1 h recycling test, a millimeter sized salt crystal can be observed on the sample surface (see the first panel in Figure 5D). Obviously, these white salt particles will introduce scattering (see Figure 5B for scanning electron microscope (SEM) image of salt crystal plates on the CP surface), which should reduce the optical absorption of the CP sample. An immediate question is whether this salt crystallization will significantly degrade the performance of the vapor generation in practice, which was not mentioned in previous reports (e.g., ref. 19, 20 performed their experiments for 1–4 h only). To clarify this issue, we then performed an 8 h continuous experiment in pure water and salt water in a beaker, respectively. Intriguingly, one can see that the evaporation speeds increased continuously and saturated at the fourth to fifth hour at ≈1.32 and ≈1.42 kg (m^2^ h)^−1^ for salt water and pure water, respectively, as shown in Figure 5C. Since the CP surface is always wet during the 8 h test (indicating sufficient water transportation contributed by capillary forces), the salt crystal did not grow further to cover the entire surface. Instead, the salt crystal area even shrank surprisingly, as shown by the photographs of the CP surface at different time spots (see Figure 5D). When we repeated this experiment (usually on the next day), this evaporation rate increase can still be observed under identical experimental conditions starting from the lower rate, indicating the stable and reusable performance for longer term seawater desalination. As shown by thermal images in Figure 5E, the average surface temperature of the CP sample increased from 44 to 45 °C gradually and saturated at 53–54 °C at the fourth to fifth hour. Therefore, the immediate next question is what introduced this surface temperature change?

According to the experimental data shown in Figures 1, 2, 3, the only observed gradual change is the bulk water temperature, as shown by dashed curves in Figure 3B. To identify this correlation, we monitored the bulk temperature over 8 h, as shown by dotted curves in Figure 5C. One can see that the bulk water temperature (from 22 to 32–33 °C) and the evaporation rate changed coincidentally. This observation demonstrated that the surface temperature of the CP‐foam is still related to the bulk liquid temperature. Due to the excellent thermal insulation of the EPS foam support employed in our structure, the temperature of the bulk water in this experiment reached the thermal equilibrium after ≈5 h. Also, due to the higher solubility of salt in warmer water, we observed that the salt crystal shrank as the bulk and surface temperature increases (i.e., Figure 5D). This vapor generation performance should be improved if better thermal insulation materials are used in the water container for small volume test. On the other hand, if the bulk water temperature change is negligible in larger scale vapor generation applications, one should not expect this obvious evaporation rate change, as will be further validated in the prototype system demonstration below.

### 2.7 A Prototype Solar Still System

**Figure 6:**
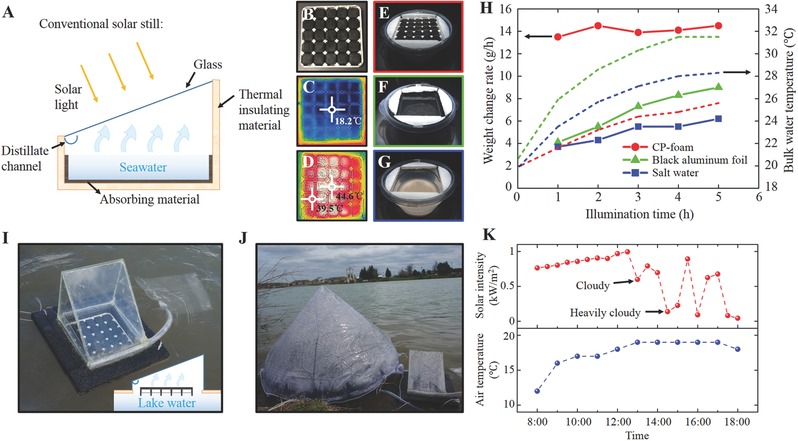
A) Schematic illustration of a conventional desalination solar still. B) Photograph of a 5 × 5 CP array with a total area of 100 cm^2^. C,D) Thermal images of CP array before (C) and after (D) solar illumination. E–G) Photographs of experimental systems with (E) the CP‐foam array on salt water, (F) bare salt water with a layer of black aluminum foil placed at the bottom, and (G) bare salt water with no CP‐foam. H) Hourly water weight change with the CP‐foam array on the water surface (red dots), black aluminum foil at the bottom (green triangles), and salt water (blue squares) as a function of illumination time. I) The photograph of a prototype system paced on Lake Lasalle at the University at Buffalo. J) The photograph of a control experiment with a commercial product (left) and our system (right) during the experiment. Obvious mist can be seen at the inner surfaces of the covers. K) The solar intensity (upper panel) and outdoor temperature curves (lower panel) from 8:00 a.m. to 6:00 p.m. on May 6, 2016 at the University at Buffalo.

A typical desalination solar still system is illustrated in **Figure** **6**A: A box made by thermal insulating materials is filled by seawater or salty water. A tilted transparent glass covers the box to collect solar light. For conventional solar vapor generation technology, light absorbing materials were usually placed at the bottom of the basin to heat the entire liquid volume with fairly low thermal efficiency (i.e., 30–40%^7^). To overcome this weakness, we developed a 5 × 5 CP array as shown in Figure 6B (i.e., 2 × 2 cm^2^ for each CP unit with the total area of 100 cm^2^), which was placed in a polypropylene box (15 cm in diameter with 1500 g water). However, thermal isolating walls have not been incorporated in this experiment. According to the thermal distribution measurement, the temperature of CP surface increased from 18.2 °C (Figure 6C under dark condition) to 44.6 °C (Figure 6D under 1 sun illumination). The slight nonuniformity of the temperature distribution in Figure 6D was introduced by the intensity distribution of the finite size of the light beam. To evaluate its performance, we repeated the solar desalination experiment using this large area sample (Figure 6E). Meanwhile, two control samples were characterized: (1) a layer of black aluminum foil placed at the bottom of the box (Figure 6F, its optical absorption spectrum is shown in Figure S4 in the Supporting Information) and (2) salty water with no CP‐foam (Figure 6G). As shown in Figure 6H, the mass change rate for the CP‐foam array is ≈1.275 kg (m^2^ h)^−1^ (with the estimated thermal efficiency η_th_ of 88.2%), which is obviously better than those for control samples (i.e., ≈0.408 kg (m^2^ h)^−1^ with η_th_ of 28.2% for the bulk heating strategy, and ≈0.242 kg (m^2^ h)^−1^ with η_th_ of 16.7% for the bare salt water evaporation). It should be noted that the evaporation rate in this large scale CP array experiment did not increase obviously. Its bulk water temperature change is also relatively small (20–25 °C, as shown by the red dashed curve in Figure 6H) due to the much larger amount of bulk water. In contrast, the evaporation rates of those two control samples increased slightly, corresponding to their bulk temperature changes, as shown by green and blue dashed curves in Figure 6H. The net water mass change produced by this 100 cm^2^ CP‐foam structure is 14.5 g after 5 h operation, which is ≈25 times of that produced by a single unit (i.e., 0.58 g h^−1^, see Figure 3). In this case, it is unnecessary to introduce a solar concentrator to enhance the water evaporation rate, which is different from the case for commercial concentrated photovoltaic systems. Due to the extremely low manufacturing cost of the CP‐foam, huge area products can easily be realized using commercial paper printing technologies at the price much lower than those for solar concentrators. Therefore, portable or large scale systems directly floating on seawater surfaces are possible to meet some low‐end freshwater generation needs, as will be demonstrated next. In this case, the costs for seawater intake and pretreatment for conventional reverse osmosis processes are largely avoided, which provides a potential solution to low‐cost freshwater generation applications.

As shown in Figure 6I, a complete portable solar still system was demonstrated by covering an open bottom box with a transparent acrylic slab (with the 0.01 m^2^ 5 × 5 CP‐foam array directly in contact with the open water below, see the inset of Figure 6I). The clean water is collected by the distillate channel and guided into a collection bag. We then placed this system on Lake Lasalle at the University at Buffalo together with a commercial solar still product with an effective area of 0.342 m^2^ (Aquamate Solar Still at the retail price of $225), as shown in Figure 6J. It should be noted that our CP‐array can take the lake water directly, while the commercial system needs to be actively fed. After a 10 h operation in the outdoor environment on a sunny–cloudy day at Buffalo with varying sunlight illumination conditions (see Figure 6K for temperature and sunlight intensity distribution), we obtained the generation productivities of 0.832 and 0.344 kg (m^2^ d)^−1^ for these two systems, respectively. The performance of the CP‐foam system is ≈2.4 times of the commercial product. In addition, due to the scattering of the mist formed on the cover (Figure 6J), the input light decreased significantly, which is the next technical issue to optimize the performance of a real system. A nontoxic superhydrophobic surface treatment for antimist on the transparent glass cover^39^ will improve the system performance, which is still under investigation but beyond the scope of this work. Consider the low‐cost of the core elements for solar‐to‐heat conversion, the solar still system can be developed at a very low‐cost (see Section S7 in the Supporting Information), which is particularly promising for the distribution in developing regions and in areas affected by natural disasters where drinking water supply is temporarily interrupted.

## 3 Conclusion

In summary, we have developed an extremely cost‐effective and efficient carbon‐based solar vapor generation system based on CP supported by floating EPS foam. Due to the efficient solar absorption of the CP, the superior thermal insulation of the EPS foam support and suppressed radiative and convective loss in the heated vapor environment, most of the absorbed solar energy is confined within a thin surface layer of liquid, resulting in efficient heat conversion and vapor generation. As a result, our system achieved a record thermal conversion efficiency of ≈88% under nonconcentrated solar illumination of 1 kW m^−2^. This corresponds to an optimized vapor generation rate that is ≈3 times greater than that of natural evaporation. In addition, stable and repeated seawater desalination tests were performed in a portable prototype both in the laboratory and an outdoor environment, and achieved a water generation rate that was 2.4 times that of a commercial product. Furthermore, by analyzing the theoretical upper limit for solar vapor generation rates, we show that the opportunity for improvement in vapor generation rates is relatively limited. This indicates that the area that offers the most potential for improvement is in the reduction of cost. Compared with previously reported advanced nanostructures, this CP–EPS system is extremely low‐cost in terms of both materials and fabrication, environmentally benign, and safe to handle during production. These attributes enable this system to be easily expanded to large scales, something that is of particular interest in regions where access to freshwater is limited. It should be noted that activated carbon structures and materials are widely used in water and gas treatment applications (e.g., ref. 40). These functionalities are inherently compatible with our CP structure, which may enable simultaneous freshwater generation and treatment from heavily contaminated source water. Considering the challenges in contaminated/waste water treatment and reuse, the development of low‐cost, electricity‐free, and multifunctional technologies represents new research avenues in carbon‐based solar vapor generation.

The shortage of freshwater and sanitation is one of the most pervasive challenges afflicting people throughout the world. It was predicted that by 2025, over half the nations in the world will face freshwater stress, and by 2050, ≈75% of the world's population could face water scarcity. Therefore, it is essential to develop technologies for disinfection and decontamination of water, and to increase water supplies through economic and sustainable ways (i.e., at lower cost, smaller energy consumption, and smaller environmental impacts). Membrane‐based separations for water purification and desalination are dominant technologies, which, unfortunately, are usually energetically demanding with serious environmental costs. There is emerging global interest in developing new technologies to address these issues. Successful demonstration of the portable solar steam generation system represents a revolutionary product to beat the conventional products both in performance and retail price, which is particularly attractive for addressing global freshwater shortages, especially in developing regions.

## Supporting information

As a service to our authors and readers, this journal provides supporting information supplied by the authors. Such materials are peer reviewed and may be re‐organized for online delivery, but are not copy‐edited or typeset. Technical support issues arising from supporting information (other than missing files) should be addressed to the authors.

Supplementary

Supplementary
