# Supplementary material for: Extremely Cost‐Effective and Efficient Solar Vapor Generation under Nonconcentrated Illumination Using Thermally Isolated Black Paper
Source: Glob Chall. 2017 Jan 30;1(2):1600003. doi: 10.1002/gch2.201600003 (PMC5445597; doi:10.1002/gch2.201600003)
Supplement: Supplementary file 1 — Supplementary [file GCH2-1-1600003-s001.pdf]

Copyright WILEY-VCH Verlag GmbH & Co. KGaA, 69469 Weinheim, Germany, 2017.

Global Challenges
Open Access

Supporting Information

for *Global Challenges*, DOI: 10.1002/gch2. 201600003

Extremely Cost-Effective and Efficient Solar Vapor Generation under Nonconcentrated Illumination Using Thermally Isolated Black Paper

*Zhejun Liu, Haomin Song, Dengxin Ji, Chenyu Li, Alec Cheney, Youhai Liu, Nan Zhang, Xie Zeng, Borui Chen, Jun Gao, Yuesheng Li, Xiang Liu, Diana Aga, Suhua Jiang, Zongfu Yu, and Qiaoqiang Gan**

Supplementary Materials for

Extremely cost-effective and efficient solar vapor generation under non-concentrated illumination using thermally isolated black paper

Zhejun Liu,[1,2,*] Haomin Song,[1,*] Dengxin Ji,[1] Chenyu Li,[1] Alec Cheney,[1] Youhai Liu,[1] Nan Zhang,[1] Xie Zeng,[1] Borui Chen,[1] Jun Gao,[2] Yuesheng Li,[2] Xiang Liu,[3] Diana Aga,[4] Suhua Jiang,[2] Zongfu Yu,[5] Qiaoqiang Gan [1,†]

1. *Department of Electrical Engineering, The State University of New York at Buffalo, Buffalo, NY 14260*
2. *Material Science Department, Fudan University, Shanghai, China 200433*
3. *Department of Environmental Science and Engineering, Fudan University, Shanghai, China 200433*
4. *Department of Chemistry, The State University of New York at Buffalo, Buffalo, NY 14260*
5. *Department of Electrical and Computer Engineering, University of Wisconsin, Madison, Wisconsin 53705, USA*

S1. CP sample preparation and stability/durability test

Sample preparation: 0.8g carbon powder (Sid Richardson Carbon & Energy Co.) was dispersed into a 160 mL water. 3 mL acetic acid was added to make carbon powder easier to attach to fibers [S1]. The mixed solution was blended well using an ultrasonic cleaner (Branson Ultrasonics Bransonic™ B200) for 5 minutes. Subsequently, the 2 cm X 2 cm white paper (Texwipe™ TX609) was put into the mixed solution to vibrate for 3 minutes so that carbon powders can dye the paper uniformly. After that, the CP was dried at 80 ºC on a heating stage. Repeat this procedure three to four times to realize the ideally dark color (see Fig. 1C in the main text).

Stability/durability test: To demonstrate the stability/durability of carbon powder attached on the paper fibers, we then cleaned a CP sample ultrasonically in clean water. We changed the water solution every 30 minutes to visualize the effect of the ultrasonic cleaning. As shown in Fig. S1A, the amount of carbon powder washed from the CP decreased gradually. After 4 hours, we cannot see obvious carbon powder in the water. Importantly, the color of the CP sample did not change obviously (Fig. S1B). To evaluate the cleaning effect of the ultrasonic vibration process, we characterized the absorption spectrum using an integration sphere spectroscopy (Thorlabs IS200-4 integrated with Ocean Optics Jaz) and confirmed that the optical performance was almost unchanged (Fig. S1C). This test provided strong evidence to demonstrate the great durability of the CP sample.

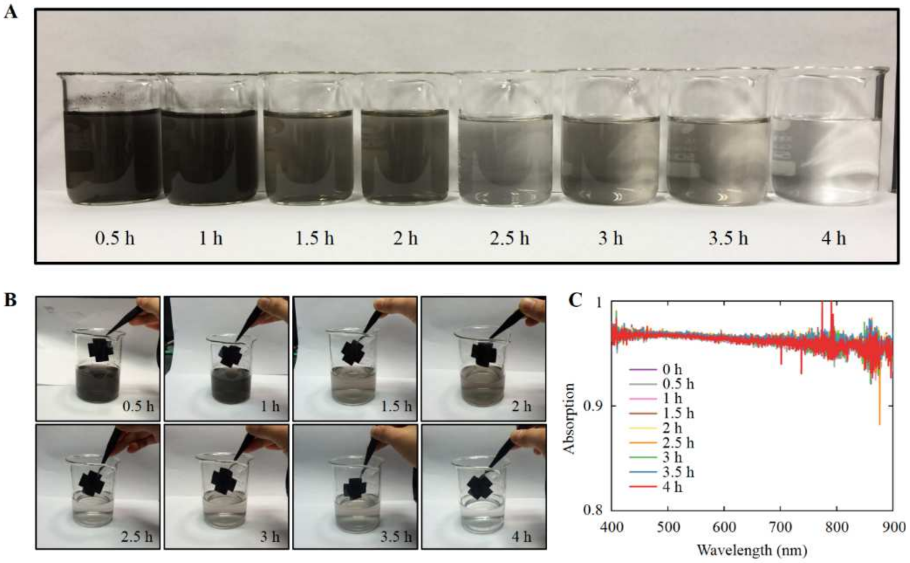

Fig. S1. (A) Color comparison of the water solution used to ultrasonically clean a CP sample after different amounts of time. (B) Photographs of the CP sample after different amounts of ultrasonic cleaning time. (C) Optical absorption spectra of the CP sample after ultrasonic cleaning.

S2. Solar vapor generation experiment setup

To measure the water evaporation rate, a 250 mL beaker filled with ~165 g water was placed under a solar simulator (Newport 69920). The CP floated on the water surface with or without the EPS foam. The residual water surface was covered by EPS foam to eliminate the natural evaporation. Two pieces of Fresnel lens (26 cm×17.8 cm, focal length: 300 mm, OpticLens) were used to concentrate solar light. 1-10 times concentrated solar light was calibrated using a powermeter (PM100D, Thorlabs Inc.) equipped with a thermal sensor (S305C, Thorlabs Inc.). The evaporation weight change was measured by an electronic scale every 10 minutes.

S3. Temperature characterization

Accurate measurement of the surface temperature is a technical challenge since it is dependent on many factors, especially the emissivity of the object being observed and the distance to the object. Therefore, thermal imager estimation of the temperature is usually not accurate, which, however, was largely neglected by most literature. To demonstrate the accuracy of the thermal imaging in our experiment, we placed two samples (i.e. a black Al foil and a CP sample) on top of a heat plate (Super-Nuova^{TM}, HP131725). When the temperature of the heat plate was set to 40 °C, the thermal image is shown in Fig. S2A. We then measured the temperature at three different positions using a thermal couple sensor probe (Signstek 6802 II, see Fig. S2B), demonstrating the reasonable accuracy of the thermal imaging (i.e., ≤0.4 °C). Therefore, the temperature change over 5-10 °C observed in the subsequent characterization is reliable based on the thermal imaging data.

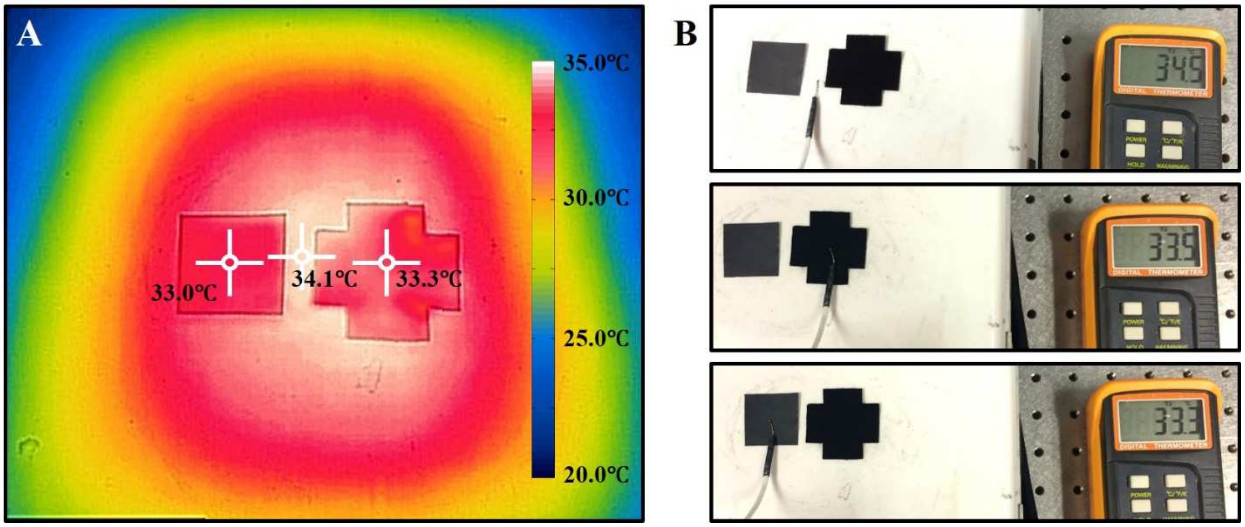

Fig. S2. (A) Surface temperature distribution of a black Al foil (left) and a CP sample (right) placed on top of a heat plate set at 40 °C. (B) Direct measurement of the temperature at three positions using a thermal couple sensor probe.

To interpret the evaporation rate difference, we employed the IR thermal imager (FLIR ONE, FLIR system) to measure the surface temperature of different samples. The vapor and liquid temperatures

were also measured by a thermometer equipped with two K-Type thermocouple sensor probes (Signstek 6802 II). One of the probes was placed above the CP sample and covered by a tiny white cardboard to eliminate the heating effect of direct illumination (Fig. S3A). The other one was placed under the CP sample to measure the temperature of bulk water (Fig S3B).

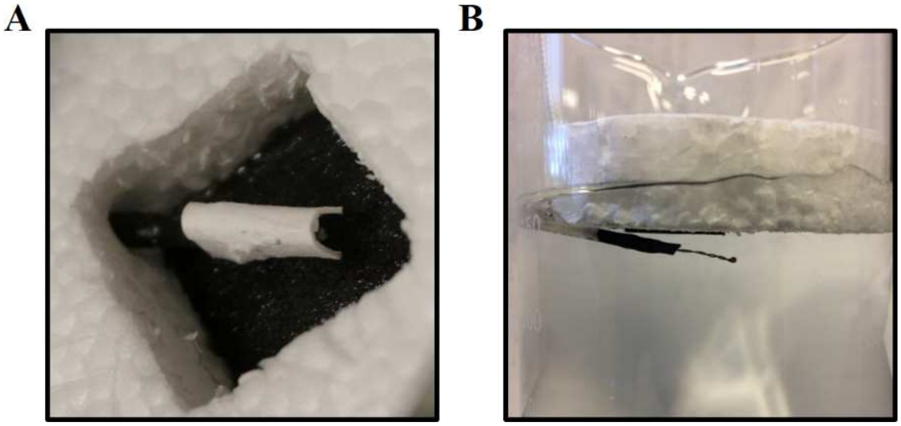

Fig. S3. Photographs of the experiment setup to measure the temperature of (A) vapor and (B) bulk water.

S4. Characterization of the liquid transportation rate of the CP

A potential concern for reduced liquid flow cross section will decrease the liquid flow rate to the CP surface. To characterize this practical upper limit, we will need to characterize the liquid transportation capability of the developed CP. Our experiment procedure is described below: We first measured the original weight of a CP sample, and then put its edge into water and employed the IR imager to monitor the water flow as the function of time (see supplementary Movie file). When the 4-cm-long sample was saturated by water in ~25 seconds, we measured the weight of the wet-CP again. In addition, one can see from the movie that the flow rate is not a constant when the paper is saturated. A more accurate characterization is under investigation and will be reported elsewhere. But by considering the small cross-sectional area of the CP-layer (i.e. ~0.2 mm X 2 cm), the practical upper limit of our CP sample is well over 1,500 kg/m^2/h, which is higher than the theoretical upper limit under 1,000X solar concentration (see the corresponding discussion in the section of “Theoretical upper limit” in the main text). Therefore, the reduced liquid flow rate is not a limitation in our current system under small to moderate solar concentration.

S5. Accurate consideration for solar thermal conversion efficiency

The thermal conversion efficiency, η_{th}, is one of the most important figures of merit to evaluate the performance of solar vapor generation, which is widely employed in all previous literature. However, the detailed values for parameters employed in those literature are slightly different. Therefore, it is necessary to explain the detail calculation to demonstrate that our obtained η_{th} is unambiguously higher than previously reported results.

The most frequently used equation for thermal conversion efficiency is $\eta_{th} = \frac{\dot{m}h_{LV}}{C_{opt}q_i}$ (i.e., eq. (1) in the main text). The variable parameter employed in different calculation is the total enthalpy of liquid-vapor phase change, h_{LV}, containing two parts: i.e., the sensible heat and the enthalpy of vaporization

(i.e., $h_{LV} = C \times (T - T_0) + \Delta h_{vap}$). In our experiments, T_0 is the initial temperature of water, i.e., 21 ℃. T is the vapor temperature measured by the thermometer, which is in the range of 40 ℃ to 90 ℃ (see data listed in Table S1). In this temperature range, the specific heat capacity of water, C, is a constant, i.e., 4.18 J/g·K. However, the enthalpy of vaporization, Δh_{vap}, is highly dependent on the temperature, which is larger at lower temperature [32]. Recent literature employed different values of h_{LV} in their calculation, resulting in certain inaccuracies in their calculated η_{th}.

For instance, Wang Y. *et al* [S2] directly employed a constant Δh_{vap} at 100 ℃ (2260 kJ/kg) as h_{LV} to calculate η_{th}. Liu Z. *et al* [18, 20] employed a temperature-dependent enthalpy of vaporization Δh_{vap}as h_{LV} to calculate η_{th}. These three literature did not consider the sensible heat (i.e., $C \times (T - T_0)$). In contrast, Ghasemi H. *et al* [22] considered the sensible heat but employed a constant Δh_{vap} at 100 ℃ (2260 kJ/kg). By considering these two terms more accurately, the solar thermal conversion efficiency of our structure under 1, 3, 5, 7, 10 times concentrated solar illumination are calculated in Table S1. Fortunately, the sensible heat (i.e., $C \times (T - T_0)$) is much smaller than Δh_{vap}, especially under small solar concentration conditions, as shown by the data listed in Table S1. Therefore, previously reported values under 1 sun illumination are still reliable but may contain up to >10% difference under 10X solar concentration.

In summary, for energy conversion efficiency estimation, the sensible heat should be considered since this energy is actually consumed by the vapor. But if one focuses on vapor generation performance, this term can be neglected since it just results in higher temperature vapor rather than generates more vapor.

Table S1. Accurate calculation of the solar thermal conversion efficiency.

Copt	$\dot{m}$ (kg/m^2·h)	Vapor temperature T (℃)	$C \times (T - T_0)$ (kJ/kg)	Δh_{vap}[32] (kJ/kg)	η_{th} (%)
1	1.28	41.6	86.1	2403.3	88.6
3	3.66	60.4	164.7	2357.6	85.5
5	6.24	69.9	204.4	2333.1	88.0
7	9.34	76.0	229.9	2320.7	94.5
10	13.30	88.9	283.8	2282.7	94.8

S6. Optical absorption of a black Al foil

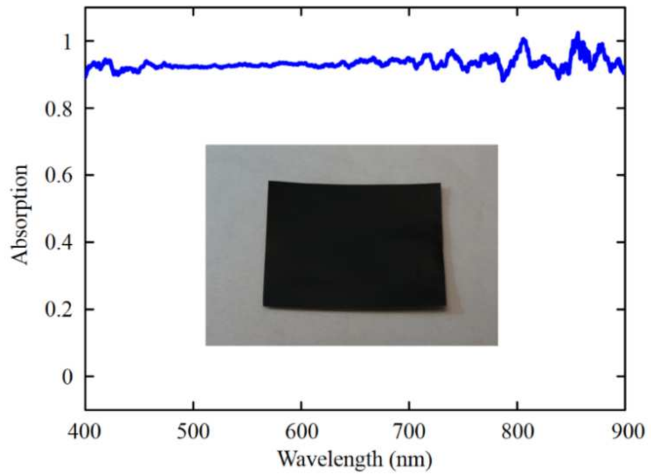

Fig. S4. Optical absorption spectrum of a black Al foil measured by an integration sphere. Inset: the photograph of a black Al foil.

S7. Cost estimation and comparison

Consider the key components for solar to heat conversion employed in all previously reported literature (e.g. metal nanoparticles or nanorods dispersed in water [11-15], metal nanoparticles on nanoporous anodic alumina [17,18,20] or airlaid paper [19], exfoliated graphite on porous carbon foam [22], porous graphene [35], a selective absorber inserted between a polystyrene foam disk and a bubble wrap [33]), the cost of our proposed structure is the lowest. In Fig. 6 of the main text, a complete system was already demonstrated using low cost plastic plates. It is well-known that the cost for plastic products are extremely low. However, the cost for condensate collection and other components are required by all solar still system, which was not discussed in recent literature. According to a review article published in 2007 [S3], the net cost of materials for conventional solar still is ~\$185.2/m^2. In contrast, our system shown in Fig. 6 is only \$76.45/m^2 based on the small scale retail price for all materials/components (see Table S2). In particular, the major cost is for the Acrylic slabs, which can be replaced by lower cost plastic boxes. Therefore, the estimated cost is lower. The net cost for mass production will be significantly lower.

Table S2. Cost of a prototype solar still system (1m^2)

	Unit price	Amount	Cost
Carbon black	\$2.26/Lb	100 g	\$0.50
Fiber-rich paper	\$1.05/m^2	1.5 m^2	\$1.58
EPS foam	\$0.59/m^3	0.5m^3	\$0.30
Acrylic slab	\$31.2/m^2	2.31 m^2	\$72.07
Collection bag	\$2/each	1	\$2
		Total	\$76.45

[S1] G. Crossmon, *Anat. Rec.* **1937**, *1*, 33.

[S2] Y. Wang, L. Zhang, P. Wang, *ACS Sustainable Chem. Eng.* **2016**, *4*,1223.

[S3] M. A. Samee, U. K. Mirza, Majeed T, et al. *Renewable and Sustainable Energy Reviews*, **2007**, *11*, 543.
